# Detection of C8/T1 radiculopathy by measuring the root motor conduction time

**DOI:** 10.1186/s12883-022-02915-8

**Published:** 2022-10-20

**Authors:** Dougho Park, Sang-Eok Lee, Jae Man Cho, Joong Won Yang, Donghoon Yang, Mansu Kim, Heum Dai Kwon

**Affiliations:** 1 Department of Rehabilitation Medicine, Pohang Stroke and Spine Hospital, 352, Huimang-daero, 37659 Pohang, Republic of Korea; 2 Department of Neurosurgery, Pohang Stroke and Spine Hospital, Pohang, Republic of Korea; 3 Department of Medical Science and Engineering, School of Convergence Science and Technology, Pohang University of Science and Technology, Pohang, Republic of Korea

**Keywords:** Radiculopathy, Electrodiagnosis, Motor-evoked potential, Nerve conduction

## Abstract

**Background:**

Root motor conduction time (RMCT) can noninvasively evaluate the status of the proximal root segment. However, its clinical application remains limited, and wider studies regarding its use are scarce. We aimed to investigate the association between C8/T1 level radiculopathy and RMCT.

**Methods:**

This was a retrospective cross-sectional study. Subjects were extracted from a general hospital’s spine clinic database. A total of 48 C8/T1 root lesions from 37 patients were included, and 48 C8/T1 root levels from control subjects were matched for age, sex, and height. RMCT was measured in the abductor pollicis brevis muscle and the assessment of any delays owing to C8/T1 radiculopathy.

**Results:**

The RMCT of the C8/T1 radiculopathy group was 1.7 ± 0.6 ms, which was significantly longer than that in the control group (1.2 ± 0.8 ms; *p* = 0.001). The delayed RMCT was independently associated with radiculopathy (adjusted odds ratio, 1.15; 95% confidence interval, 1.06–1.27; *p* = 0.011) after adjusting for the peripheral motor conduction time, amplitude of median compound motor nerve action potential, and shortest F-wave latency. The area under the Receiver Operating Characteristic curve for diagnosing C8/T1 radiculopathy using RMCT was 0.72 (0.61–0.82). The RMCT was significantly correlated with symptom duration (coefficient = 0.58; *p* < 0.001) but was not associated with the degree of arm pain.

**Conclusion:**

Our findings illustrate the clinical applicability of the RMCT by demonstrating its utility in diagnosing radiculopathy at certain spinal levels.

**Supplementary information:**

The online version contains supplementary material available at 10.1186/s12883-022-02915-8.

## Introduction

Radiculopathy is a common condition whose symptoms can include pain, sensory change, and motor weakness owing to mechanical and chemical irritation of the spinal nerve root [1, 2]. Imaging studies are essential for diagnosing radiculopathy; magnetic resonance imaging helps identify soft tissues such as discs, ligaments, and nerve roots [3], while computed tomography is primarily used to confirm abnormalities in bony structures [4]. Meanwhile, another key factor in confirming radiculopathy is electrophysiologic diagnosis [5]. In particular, electromyography (EMG) is a classical tool that was used to evaluate radiculopathy before the development of modern imaging instruments [6]; its key advantage is in its ability to detect neurophysiological changes that cannot be confirmed by imaging studies alone [7]. By applying EMG to multiple myotomes, it is possible not only to localize the abnormal spinal root level but also to estimate the temporal aspect of compression by distinguishing motor unit action potential [8]. However, the invasive nature of EMG creates a potential risk of complications such as bleeding or infection at the examination site [9]. Additionally, patient cooperation is essential for observing resting potential and interference patterns [10].

Root motor conduction time (RMCT) is a measure of the state of the proximal root segment that is obtained noninvasively by performing direct cervical stimulation and nerve conduction studies [11, 12]. Notably, RMCT is very localized since it excludes distal neural lesions and measures only the conduction time of the proximal root segment [13–15]. However, it also has the disadvantage of involving a somewhat complicated measurement method wherein well-trained examiners are required [16]. To date, the RMCT has been investigated in only a few disease types, such as demyelinating disease and lumbar spinal stenosis, whereas its clinical applicability in patients with radiculopathy remains unclear [17].

In this study, we hypothesized that the RMCT can be a valuable tool for the diagnosis of radiculopathy at certain spinal root levels. To that end, we investigated whether the RMCT measured in the abductor pollicis brevis (APB) muscle was significantly delayed in the presence of C8/T1 radiculopathy. Moreover, we aimed to determine the association between clinical symptoms and RMCT.

## Methods

### Subjects and clinical evaluations

We performed a retrospective cross-sectional study based on medical records of patients treated between June 2007 and December 2021. We extracted patients diagnosed with C8/T1 radiculopathy at our hospital through electrodiagnosis and whose RMCT was measured at the same time. All patients diagnosed with C8/T1 radiculopathy also underwent cervical x-ray and magnetic resonance imaging studies to confirm root compression and exclude other differential diagnoses. Thereafter, the final study group was selected by applying the following exclusion criteria: uncontrolled diabetes, concomitant polyneuropathy or median nerve lesion, previous cervical spine surgery, previous hand injury or surgery, unobtainable electrodiagnostic parameters, and lack of clinical information. Data from 48 C8/T1 root lesions in 37 patients were finally extracted; these included the subjective symptom duration, numerical rating scale (NRS) of the neck and arm pain, and neck disability index (NDI) score at the time of electrodiagnostic examination.

**Fig. 1:**
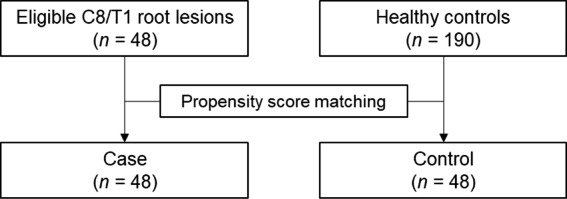
Flowchart showing the comparative analysis of the patients and control subjects

The control group consisted of a single-center healthy cohort of 190 prospectively analyzed subjects who were reported previously by the authors [18]. We controlled the potential biases between the two groups by performing propensity score matching (PSM). Consequently, 96 root levels (48 patients and 48 matched controls) were finally investigated (Fig. 1).

This study was approved by the Institutional Review Board of Pohang Stroke and Spine Hospital (PSSH0475-202207-HR-011-01); informed consent was waived, given its retrospective design. The research complied with the guidelines of the Declaration of Helsinki.

### Electrodiagnostic assessments

The EMG protocol for radiculopathy comprised of the following [5]: First, we examined muscles innervated by different nerves in the same myotome. Second, we assessed both proximal and distal muscles in the same myotome. Finally, we examined muscles in the myotomes adjacent to the suspected level. EMG-confirmed C8/T1 radiculopathy was defined as the presence of denervation potentials or polyphasic, long-duration, and large-amplitude motor unit action potentials in two or more of the differently innervated following muscles after other conditions were ruled out: flexor pollicis longus, flexor digitorum profundus, flexor carpi radialis, first dorsal interosseous, abductor digiti minimi, and APB [5].

For the nerve conduction study, we first recorded the sensory nerve action potentials of the median, ulnar, and superficial radial nerve in the distal arm. Electrical stimulations with 0.1 ms square wave pulses were applied for the sensory nerve conduction studies with a 10–2000 Hz filter setting. Next, we recorded compound motor nerve action potentials from the APB and abductor digiti minimi muscles and F-waves from the APB muscle. To conduct motor nerve conduction studies, each nerve was supramaximally stimulated by 0.2 ms square wave pulses with a 5–5000 Hz filter setting. We recorded nerve action potentials at least 12 times at the same nerve for study reproducibility. Surface electrodes were attached using the belly-tendon method to all recorded muscles. All nerve conduction studies were performed in the supine position. The detailed methods for individual nerve conduction studies are described in Supplementary Table 1 [5, 19].

**Fig. 2:**
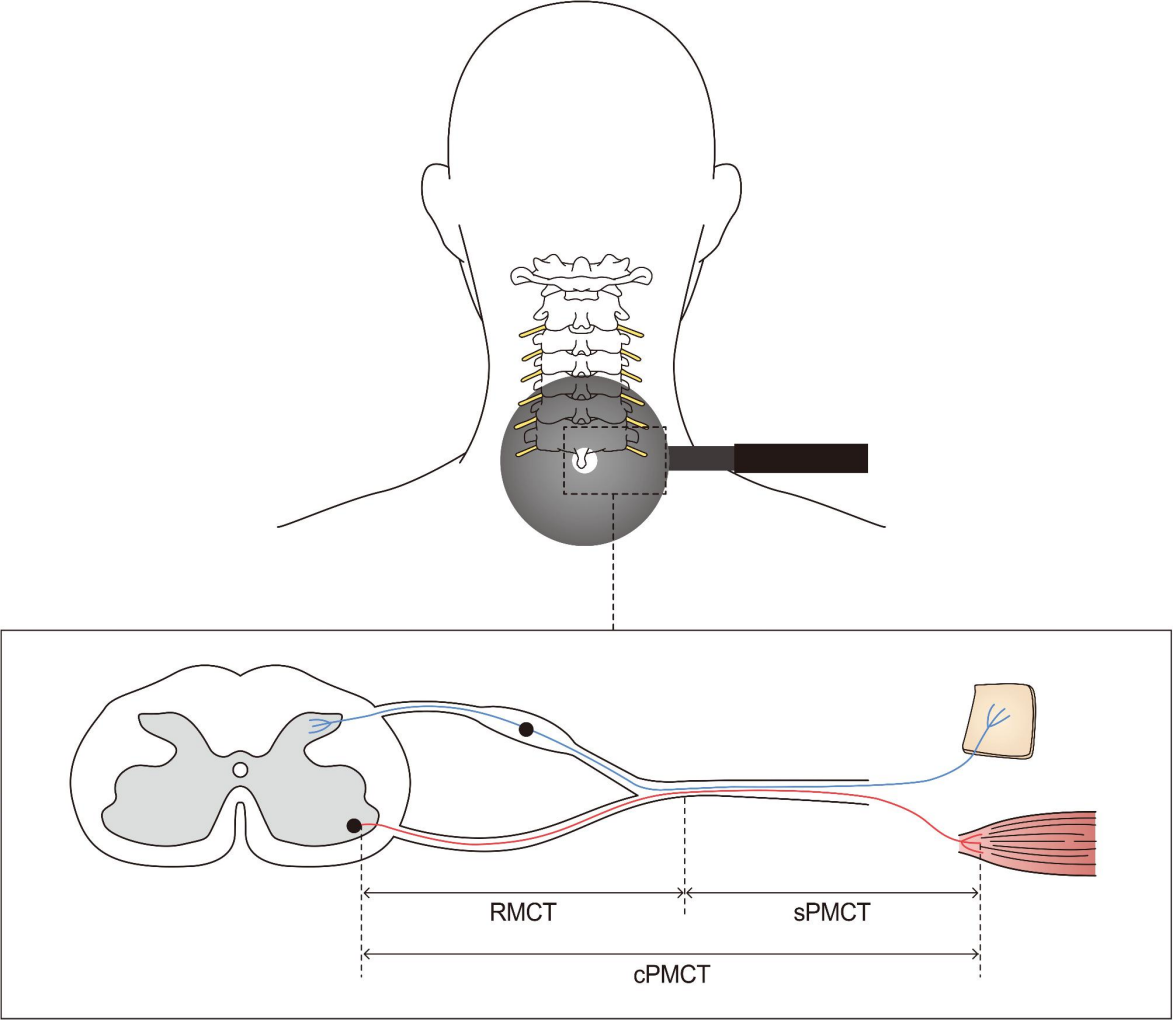
Schematic diagram illustrating the measurement of C8/T1 root motor conduction time

To measure the stimulated peripheral motor conduction time (PMCT), we performed magnetic stimulation at the C7 spinous process; the ABP muscle was recorded using the surface electrodes. To provoke median motor evoked potential, we administered supramaximal stimulation (20% above the threshold) with weak isometric contraction in the APB muscle. The simulations were applied biphasic and the active pulse width was 280 µs. Then, we calculated the RMCT using the following equations, which are based on the principle that nerve excitement occurs a few centimeters distal to the anterior horn cell upon magnetic stimulation (Fig. 2) [20].

RMCT (ms) = calculated PMCT − stimulated PMCT

Calculated PMCT (ms) = (compound muscle action potential onset latency + F-wave onset latency − 1) / 2

Stimulated PMCT (ms) = spinal motor-evoked potential onset latency

All electrodiagnostic evaluations were performed using the Cadwell Sierra Wave EMG system (Cadwell Laboratories Inc., Kennewick, WA, USA). A MagPro Compact with a C-100 circular coil (11 cm outer diameter) (MagVenture Inc, Farum, Denmark) was used for cervical magnetic stimulation. The temperature of the electrodiagnostic laboratory was maintained at approximately 23℃ to 25℃. All electrodiagnostic examinations were interpreted by experienced physiatrists.

### Statistical analysis

The Shapiro-Wilk test was applied to check the normality of continuous variables. These are expressed as means ± standard deviations if normality was satisfied or as medians (interquartile ranges) if not; the independent t-test and Mann-Whitney U-test were applied for comparative analyses of these types of values, respectively. Categorical variables are expressed as frequencies (proportions); groups were compared using the chi-squared test. We established binary logistic regression models for detecting C8/T1 root lesions using data adjusted for age, sex, and height. Model 1 was a predictive model of RMCT alone, Model 2 was adjusted for the stimulated PMCT, and Model 3 was adjusted for the median compound muscle action potential and F-wave in addition to the adjustment of Model 2. The multicollinearity of the model was confirmed, as the variance inflation factor was < 10. We drew the Receiver Operating Characteristic curve and calculated the cutoff value using Youden’s J statistic. We calculated the Spearman coefficient to determine the correlation between RMCT and symptoms. All statistical analyses were performed using SPSS 22.0 (IBM Inc., Armonk, NY, USA).

For PSM, the “MatchIt” package of the R software version 4.1.2 (R Core Team, R Foundation for Statistical Computing, Vienna, Austria) was used [21]. The covariates used for matching were age, sex, and height; moreover, 1:1 matching with no replacement was performed. The nearest-neighbor method was applied with the caliper set to 0.2.

## Results

### Baseline characteristics of patients and controls

Table 1 summarizes the baseline characteristics of the 37 patients, 11 of whom had bilateral C8/T1 radiculopathy. The mean patient age was 61.1 years and the mean height was 166.5 cm; 75.7% were men. The patients’ median NDI score was 12.0. Among all 48 C8/T1 root levels, 25 (52.1%) were right-sided, and the median duration of symptoms was 4.0 (3.0–12.0) months. The median NRS scores of the neck and arm were 3.0 and 4.5, respectively.

**Table 1:** Baseline features of patients with C8/T1 level radiculopathy

Variables	Value^a^
Total patients, *n*	37
Age, years	61.1 ± 12.2
Male, *n* (%)	28 (75.7)
Height, cm	166.5 ± 8.7
Hypertension, *n* (%)	16 (43.2)
Diabetes, *n* (%)	5 (13.5)
Dyslipidemia, *n* (%)	6 (16.2)
NRS, neck	3.0 (2.0–4.5)
Total C8/T1 root levels, *n*	48
Right side, *n* (%)	25 (52.1)
Symptom duration, months	4.0 (3.0–12.0)
NDI	12.0 (5.5–19.0)
NRS, arm	4.5 (3.0–6.0)

Abbreviations: NDI, neck disability index; NRS, numerical rating scale

^a^Unless otherwise indicated, values are means ± standard deviations or medians (interquartile ranges)

After performing PSM for comparative analysis, we extracted 48 control C8/T1 root levels and confirmed that there were no significant differences in age, sex, or height between the two groups (Table 2).

**Table 2:** Comparison of data from patients with C8/T1 level radiculopathy and matched control subjects

	Patients (*n* = 48)	Controls (*n* = 48)	*p*-value
Age, years	61.9 ± 12.4	58.8 ± 11.1	0.197
Male, *n* (%)	37 (77.1)	28 (58.3)	0.081
Height, cm	166.4 ± 8.6	164.2 ± 8.8	0.223
APB-RMCT, ms	1.7 ± 0.6	1.2 ± 0.8	0.001

Abbreviations: APB, abductor pollicis brevis; RMCT, root motor conduction time

### RMCT at the C8/T1 level

**Fig. 3:**
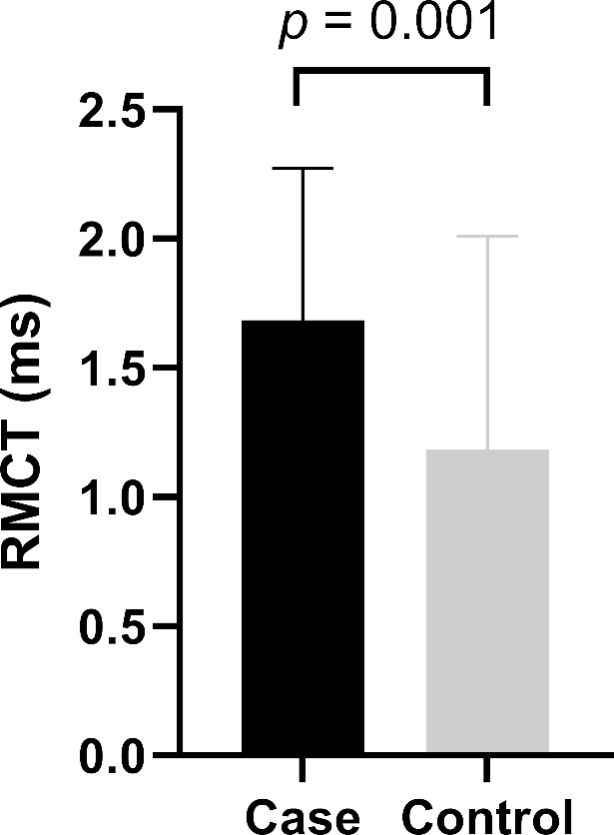
Root motor conduction time (RMCT) in each group. Patients with C8/T1 level radiculopathy showed significantly longer RMCTs than did the control group (*p* = 0.001)

The mean RMCT among patients with radiculopathy was 1.7 ± 0.6 ms, which was significantly longer than that among control subjects (1.2 ± 0.8 ms; *p* = 0.001) (Table 2; Fig. 3). Logistic regression models revealed that the delay in RMCT observed in patients with C8/T1 was independently associated with radiculopathy (per Model 3: odds ratio, 1.15; 95% confidence interval, 1.06–1.27; *p* = 0.011) (Table 3). The area under the Receiver Operating Characteristic curve for diagnosing C8/T1 radiculopathy using RMCT was 0.72 (0.61–0.82) with 0.83 sensitivity and 0.58 specificity (Supplementary Fig. 1). In terms of subjective symptom indices, the RMCT was significantly associated with symptom duration (coefficient = 0.58; *p* < 0.001) but not with the NRS of the arm (coefficient = 0.22; *p* = 0.139) All measured values for calculating the RMCT are presented in Supplementary Table 2.

**Table 3:** Logistic regression models using data from patients and matched control subjects

		Odds ratio (95% CI)	*p*-value
Model 1^a^	RMCT (per 0.1 ms)	1.11 (1.04–1.18)	0.002
Model 2^b^	RMCT (per 0.1 ms)	1.16 (1.06–1.27)	0.002
Model 3^c^	RMCT (per 0.1 ms)	1.15 (1.06–1.27)	0.011

Abbreviations: CI, confidence interval; RMCT, root motor conduction time

^a^Univariable analysis; ^b^adjusted for stimulated peripheral motor conduction time; ^c^adjusted for stimulated peripheral motor conduction time, amplitude of median compound motor nerve action potential, and F-wave latency

## Discussion

We observed a significant delay in RMCT among patients with C8/T1 radiculopathy when compared to control subjects, and also demonstrated that this delay was independently associated with the presence of radiculopathy at the C8/T1 level. This finding is notable because it provided a rational basis for the applicability of RMCT in terms of diagnosing radiculopathy at a specific spinal root level.

The application of RMCT to assess radiculopathy occurring at particular levels is rare. Most relevant studies were conducted before the 2000s, with only a few published since then. Banerjee et al. [13] described magnetic spinal stimulation as a non-invasive method to evaluate lumbosacral motor radiculopathy in 26 patients and 25 control subjects; they mainly targeted the lower lumbosacral nerve roots (L5, S1, and S2) by recording from the abductor hallucis muscle. According to their results, patients with clinical motor weakness had profoundly prolonged RMCTs; therefore, they suggested that RMCT delay was related to the severity of symptoms. Golez [22] measured motor conduction time via stimulation at the L1 and S1 levels in 25 patients with lumbar spinal stenosis and 36 control subjects. That study was noteworthy because it demonstrated that neurogenic claudication increased the cauda equina motor conduction time, revealing that the clinical symptom and delay of motor conduction time were related. In our study, the RMCT showed a significant correlation with the duration of subjective symptoms, which supports Golez’s results to some extent. However, in our study, the subjective pain level in the lesional-side limb was not related to RMCT as we assumed when we primarily evaluated the motor nerve. Additionally, Seçil et al. [23] measured the cauda equina motor conduction time and showed that it was slower in the lumbar spinal stenosis group than in the control group; however, their study did not identify the relationship between the degree of motor conduction delay and symptom severity.

Our study is the first to measure RMCT in patients with cervical radiculopathy and has the advantage of being specific to a specific spinal level; moreover, we included a relatively large number of subjects compared to previous related studies. Additionally, variables such as age, sex, and height were analyzed and adjusted for using PSM, rendering the results much more reliable. Since the APB-RMCT was previously found to be affected by height in a linear model [18], for the integrity of our results, it was important that we controlled for this factor when assessing the patient and control groups.

RMCT has also been studied in patients with demyelinating neuropathy. In a previous study, a significant difference was found in the RMCT of 30 healthy subjects and 12 patients with Guillain-Barre syndrome who were diagnosed within one week [14]; hence, RMCT was deemed to be useful for the early diagnosis of focal segmental demyelinating polyneuropathy, affording this measure a novel clinical utility. In the study of Inaba et al. [24], RMCT was measured in 11 patients with chronic inflammatory demyelinating neuropathy and 10 with Guillain-Barre syndrome, wherein the authors found that the RMCT increasingly normalized as muscle strength recovered. Taken together, such studies illustrate the applicability of the RMCT to various diseases involving the motor root segment. Furthermore, measuring the RMCT is a non-invasive procedure and has the potential to be applied to additional fields given its ability to detect diseases in their early stages and to also assess functional recovery. Therefore, additional research on the use of the RMCT is warranted going forward.

This study had several limitations. It was retrospective in nature, requiring additional investigations to validate our results. Additionally, our participants were limited to Koreans; hence, validation among patients of other ethnicities is also required. Our patients comprised a single group diagnosed with C8/T1 radiculopathy that was relatively heterogeneous; future studies that analyze various subgroups of patients (such as those with acute versus chronic lesions or mild versus severe symptoms), would further clarify the relationship between RMCT and radiculopathy. Finally, there were potential limitations in terms of measurement. For example, it is difficult to apply our methods at some spinal root levels because reliable recording of the F-wave is possible only from distal muscles [25]. Moreover, it was challenging to measure the RMCT when reliable motor evoked potentials or compound muscle action potentials could not be obtained owing to severe peripheral nerve lesions in the distal limbs.

## Conclusion

We demonstrated that the RMCT is delayed in the root lesions of patients with C8/T1 level radiculopathy. This noninvasive method (compared to EMG) can have an adjuvant role in diagnosing radiculopathy at certain spinal levels. Our study may be a good milestone for future clinical applications of RMCT as related to patients with radiculopathy. However, it is important to validate our results through additional multi-center, multi-ethnic studies.

## Electronic supplementary material

Below is the link to the electronic supplementary material.

Supplementary Material 1

## Data Availability

The dataset supporting the conclusions of this article is included within the article and its additional files.

## Abbreviations

ABP: abductor pollicis brevis
EMG: electromyography
NDI: neck disability index
NRS: numerical rating scale
PMCT: peripheral motor conduction time
PSM: propensity score matching
RMCT: root motor conduction time.

## References

[1] Tarulli AW, Raynor EM (2007). Lumbosacral radiculopathy. Neurol Clin.

[2] Mansfield M, Smith T, Spahr N, Thacker M (2020). Cervical spine radiculopathy epidemiology: A systematic review. Musculoskelet Care.

[3] Reza Soltani Z, Sajadi S, Tavana B (2014). A comparison of magnetic resonance imaging with electrodiagnostic findings in the evaluation of clinical radiculopathy: a cross-sectional study. Eur Spine J.

[4] Mena J, Sherman AL (2011). Imaging in radiculopathy. Phys Med Rehabil Clin N Am.

[5] Dumitru D, Amato AA, Zwarts MJ (2002). Electrodiagnostic medicine.

[6] Rubinstein SM, Pool JJ, van Tulder MW, Riphagen II, de Vet HC (2007). A systematic review of the diagnostic accuracy of provocative tests of the neck for diagnosing cervical radiculopathy. Eur Spine J.

[7] Li W, Liu YC, Zheng CF, Miao J, Chen H, Quan HY, Yan SH, Zhang K (2018). Diagnosis of Compressed Nerve Root in Lumbar Disc Herniation Patients by Surface Electromyography. Orthop Surg.

[8] Tamarkin RG, Isaacson AC. Electrodiagnostic Evaluation Of Lumbosacral Radiculopathy. In: StatPearls. edn. Treasure Island (FL); 2022. 33085371

[9] Cushman DM, Strenn Q, Elmer A, Yang AJ, Onofrei L (2020). Complications Associated With Electromyography: A Systematic Review. Am J Phys Med Rehabil.

[10] Chemali KR, Tsao B (2005). Electrodiagnostic testing of nerves and muscles: when, why, and how to order. Cleve Clin J Med.

[11] Matsumoto H, Hanajima R, Terao Y, Ugawa Y (2013). Magnetic-motor-root stimulation: review. Clin Neurophysiol.

[12] Mills KR, McLeod C, Sheffy J, Loh L (1993). The optimal current direction for excitation of human cervical motor roots with a double coil magnetic stimulator. Electroencephalogr Clin Neurophysiol.

[13] Banerjee TK, Mostofi MS, Us O, Weerasinghe V, Sedgwick EM (1993). Magnetic stimulation in the determination of lumbosacral motor radiculopathy. Electroencephalogr Clin Neurophysiol.

[14] Temucin CM, Nurlu G (2011). Measurement of motor root conduction time at the early stage of Guillain-Barre syndrome. Eur J Neurol.

[15] Rayegani SM, Hollisaz MT, Hafezi R, Nassirzadeh S (2008). Application of magnetic motor stimulation for measuring conduction time across the lower part of the brachial plexus. J Brachial Plex Peripher Nerve Inj.

[16] Samii A, Luciano CA, Dambrosia JM, Hallett M (1998). Central motor conduction time: reproducibility and discomfort of different methods. Muscle Nerve.

[17] Weber M, Eisen AA (2002). Magnetic stimulation of the central and peripheral nervous systems. Muscle Nerve.

[18] Park D, Kim BH, Lee S-E, Cho JM, Yang JW, Yang D, Kim M, Oh G, Sophannara Y, Kwon HD. Normal values of central, peripheral, and root motor conduction times in a healthy Korean population. J Clin Neurophysiol 2022, Publish Ahead of Print. 10.1097/WNP.0000000000000954 38306225

[19] Preston DC, Shapiro BE, Bolck F (2005). Electromyography and Neuromuscular Disorders: Clinical-Electrophysiologic Correlations.

[20] Chokroverty S, Picone MA, Chokroverty M (1991). Percutaneous magnetic coil stimulation of human cervical vertebral column: site of stimulation and clinical application. Electroencephalogr Clin Neurophysiol.

[21] Zhao QY, Luo JC, Su Y, Zhang YJ, Tu GW, Luo Z (2021). Propensity score matching with R: conventional methods and new features. Ann Transl Med.

[22] Golez A. Motor conduction time along the cauda equina at rest and after walking following electrical and magnetic stimulation. J Surg Res 2020, 03(01).

[23] Secil Y, Ekinci AS, Bayram KB, Incesu TK, Tokucoglu F, Gurgor N, Ozdemirkiran T, Basoglu M, Ertekin C (2012). Diagnostic value of cauda equina motor conduction time in lumbar spinal stenosis. Clin Neurophysiol.

[24] Inaba A, Yokota T, Otagiri A, Nishimura T, Saito Y, Ichikawa T, Mizusawa H (2002). Electrophysiological evaluation of conduction in the most proximal motor root segment. Muscle Nerve.

[25] Fisher MA (2007). F-waves–physiology and clinical uses. ScientificWorldJournal.

