# Supplementary material for: Detection of C8/T1 radiculopathy by measuring the root motor conduction time
Source: BMC Neurol. 2022 Oct 20;22:389. doi: 10.1186/s12883-022-02915-8 (PMC9583482; doi:10.1186/s12883-022-02915-8)
Supplement: Supplementary file 1 — Supplementary Material 1 [file 12883_2022_2915_MOESM1_ESM.pdf]

Supplementary Table 1. Detailed methods for electrodiagnosis in this study [1, 2].

Studies	Nerves	Stimulation site	Recording site	Distal distance	Direction
SNAP	Median	Middle of the wrist	2nd finger	14 cm	Antidromic
	Ulnar	Medial wrist next to the FCU tendon	5th finger	12 cm	Antidromic
	Radial	Over the distal-mid radius	E1: over the extensor tendon of the thumb<br>E2: 3–4cm distal to E1	10cm	Antidromic
CMAP	Median	Proximal: antecubital fossa<br>Distal: middle of the wrist	APB muscle	7 cm	Orthodromic
	Ulnar	Proximal: 3–4cm distal to the medial epicondyle<br>Distal: medial wrist next to the FCU tendon	ADM muscle	7 cm	Orthodromic
F-wave	Median	Middle of the wrist	APB muscle	7 cm	Antidromic

ADM, abductor digiti minimi; APB, abductor pollicis brevis; CMAP, compound motor nerve action potential; FCU, flexor carpi ulnaris; SNAP, sensory nerve action potential.

References

1. Dumitru D, Amato AA, Zwarts MJ: Electrodiagnostic medicine, 2nd edn. Philadelphia: Hanley & Belfus; 2002.

2. Preston DC, Shapiro BE: Electromyography and Neuromuscular Disorders: Clinical-Electrophysiologic Correlations. 2nd edn. Philadelphia, PA: Elsevier Butterworth-Heinemann, 2005.

Supplementary Figure 1. Receiver operating characteristic curve of root motor conduction time for detecting C8/T1 radiculopathy.

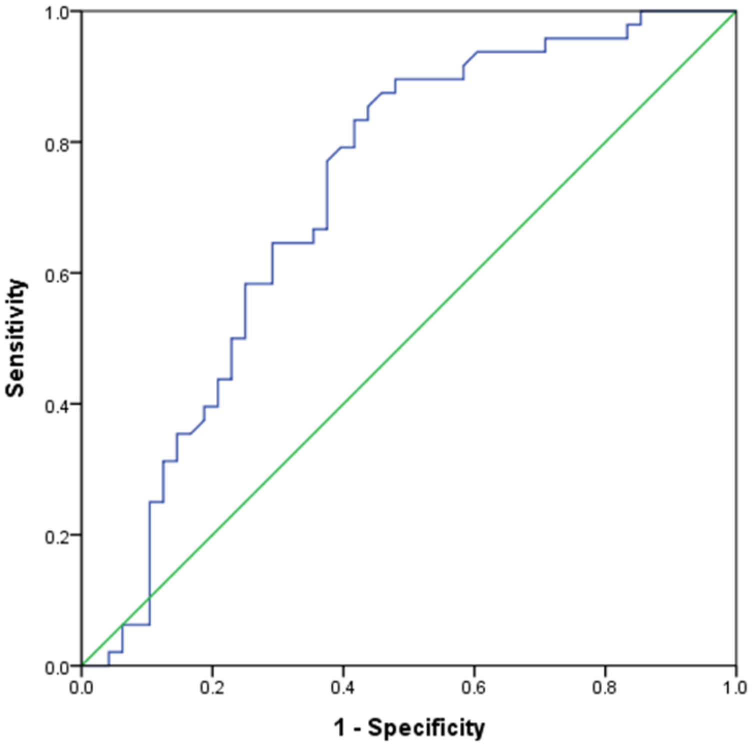

Cutoff value (seconds)	1.13
Area under the curve	0.719 (0.614–0.824)
Sensitivity	0.83
Specificity	0.58
Positive predictive value	0.67
Negative predictive value	0.22

Supplementary Table 2. Measured values related to root motor conduction time.

	Case (n = 48)	Control (n = 48)	p-value
APB-cervical MEP latency, ms	13.5 ± 1.1	13.2 ± 1.1	0.107
Median CMAP latency, ms	3.7 ± 0.5	3.3 ± 0.4	<0.001
F-wave latency, ms	27.8 ± 1.9	26.4 ± 1.7	<0.001

APB, abductor pollicis brevis; CMAP, compound motor nerve action potential; MEP, motor evoked potential.
